# Selective Calpain Inhibition Improves Functional and Histopathological Outcomes in a Canine Spinal Cord Injury Model

**DOI:** 10.3390/ijms231911772

**Published:** 2022-10-04

**Authors:** Elsayed Metwally, Hatim A. Al-Abbadi, Mohamed A. Hashem, Yasmina K. Mahmoud, Eman A. Ahmed, Ahmed I. Maaty, Ibrahim E. Helal, Mahmoud F. Ahmed

**Affiliations:** 1 Department of Cytology and Histology, Faculty of Veterinary Medicine, Suez Canal University, 4.5 Ring Road, Ismailia 41522, Egypt; 2 Faculty of Medicine, University Hospital, King Abdulaziz University, Jeddah 80212, Saudi Arabia; 3 Department of Surgery, Anesthesiology and Radiology, Faculty of Veterinary Medicine, Suez Canal University, 4.5 Ring Road, Ismailia 41522, Egypt; 4 Department of Biochemistry, Faculty of Veterinary Medicine, Suez Canal University, 4.5 Ring Road, Ismailia 41522, Egypt; 5 Department of Pharmacology, Faculty of Veterinary Medicine, Suez Canal University, 4.5 Ring Road, Ismailia 41522, Egypt; 6 Department of Physical Medicine, Rheumatology and Rehabilitation, Faculty of Medicine, Suez Canal University, 4.5 Ring Road, Ismailia 41522, Egypt

**Keywords:** calpain, calpain inhibitor, cBBB, dogs, methylprednisolone sodium succinate, neurodegeneration, somatosensory evoked potential, spinal cord injury

## Abstract

Calpain activation has been implicated in various pathologies, including neurodegeneration. Thus, calpain inhibition could effectively prevent spinal cord injury (SCI) associated with neurodegeneration. In the current study, a dog SCI model was used to evaluate the therapeutic potential of a selective calpain inhibitor (PD150606) in combination with methylprednisolone sodium succinate (MPSS) as an anti-inflammatory drug. SCI was experimentally induced in sixteen mongrel dogs through an epidural balloon compression technique. The dogs were allocated randomly into four groups: control, MPSS, PD150606, and MPSS+PD150606. Clinical evaluation, serum biochemical, somatosensory evoked potentials, histopathological, and immunoblotting analyses were performed to assess treated dogs during the study. The current findings revealed that the combined administration of MPSS+PD150606 demonstrated considerably lower neuronal loss and microglial cell infiltration than the other groups, with a significant improvement in the locomotor score. The increased levels of inflammatory markers (GFAP and CD11) and calcium-binding proteins (Iba1 and S100) were significantly reduced in the combination group and to a lesser extent in MPSS or PD150606 treatment alone. Interestingly, the combined treatment effectively inhibited the calpain-induced cleavage of p35, limited cdk5 activation, and inhibited tau phosphorylation. These results suggest that early MPSS+PD150606 therapy after acute SCI may prevent subsequent neurodegeneration via calpain inhibition.

## 1. Introduction

A severe spinal cord injury (SCI) that results in the complete loss of sensory and motor functions is one of the gravest neurological conditions in both dogs and humans. The loss of movement suddenly after SCI is life-changing and serves as a primary motivator for research into the development of novel therapeutic strategies [1,2]. Primarily, SCI is caused by mechanical damage, which results in immediate physical and biochemical abnormalities. SCI disrupts the nerve connections that link the brain and the body, resulting in paraparesis or paralysis [3]. Following these changes, a secondary injury mechanism is initiated, which includes a range of cellular, vascular, and biochemical events [4,5]. SCI is often accompanied by the formation of a hematoma as well as oxidative and inflammatory responses [6]. This process is manifested by mediator release, enzyme activation, inflammatory cell migration, glial activation, and neural tissue degradation that occur within hours or days after injury [7,8,9].

Various animal models have been widely developed to investigate the pathological consequences of SCI and establish an effective treatment basis [10,11]. The canine SCI model has recently gained attention as a translational model for confirming and extending results from rodent investigations of SCI therapies [12,13]. In addition, unlike many rodents, severe SCI in dogs does not show a remarkable spontaneous recovery [14]. Dogs have been used in SCI research as they have histopathologic similarities to human patients [15]. Furthermore, neurological examinations and full pathophysiological assessments are easier to conduct on dogs [1,16].

Calpain activation is widely involved in neurodegeneration such as brain and spinal cord damage in both humans and animals [17,18]. Considerable SCI research is focused on discovering strategies to prevent or avoid the ensuing damage caused by enzyme activation, such as the calcium-dependent protease calpain. Several lines of evidence in various models and humans highlight the need to investigate the potential therapeutic efficacy of calpain inhibitors in neurodegenerative disorders and other systemic diseases [18,19,20]. Calpain activation leads to the cleavage of p35 to p25, which contributes to the prolonged activation and mislocalization of cyclin-dependent kinase 5 (cdk5), resulting in neuronal death. Deregulated cdk5 also promotes neuronal death by hyperphosphorylating tau [21,22]. Therefore, selective calpain inhibitors seem promising as therapeutic alternatives for acute SCI-induced neurodegeneration.

The neuroprotective methylprednisolone sodium succinate (MPSS) has been regarded as a “standard of care” in both veterinary and human treatment of SCI [14,16,23]. It has been investigated in an array of medical studies for its ability to minimize free radical formation, spinal cord ischemia, and inflammation as well as lower the level of cell membrane lipid peroxidation, hence minimizing secondary damage following SCI [16,23]. High dosages of MPSS, on the other hand, induce plenty of complications, including an increased risk of infection, gastrointestinal disturbances, pneumonia, pressure sores, and deep vein thrombosis [24]. On the other hand, recent cohort studies have shown no marked difference in clinical outcomes between SCI patients treated with and without MPSS [16,25].

For the first time to our knowledge, the current study aimed to examine the role of the calpain inhibitor PD150606 in combination with MPSS as a neuroprotective drug in an induced acute SCI model in dogs. We evaluated PD150606 and MPSS, and their combination by comparing their anti-inflammatory activity, calpain inhibition, and functional recovery.

## 2. Results

### 2.1. Clinical Evaluation

**Figure 1:**
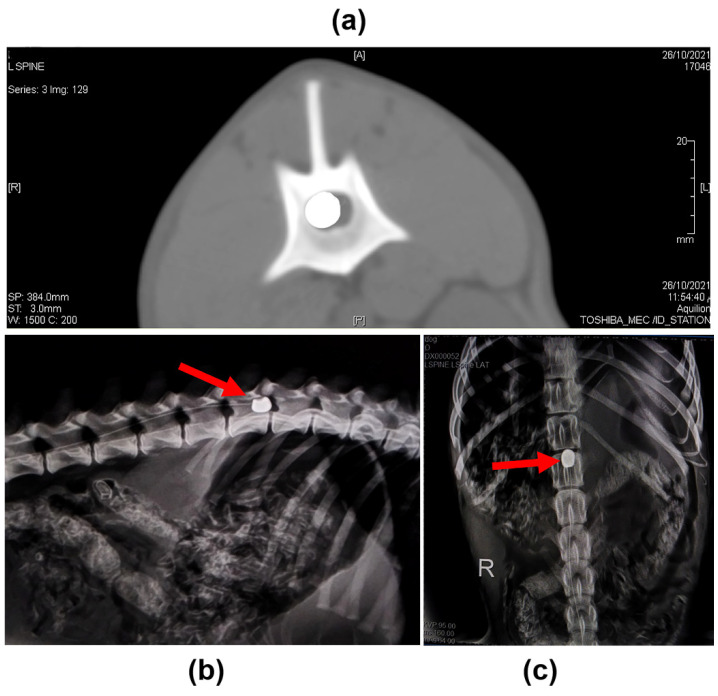
SCI induction using the balloon compression approach. (**a**) Transverse CT image at the level of L1 after balloon inflation. (**b**) Plain radiography (lateral view) after balloon inflation at the level of L1 (red arrow). (**c**) Plain radiography (Dorso-ventral view) of the same image is shown in (**b**) (red arrow).

The balloon compression catheter revealed spinal canal occlusion using radiography and computed tomography (CT) (Figure 1). The clinical state after SCI induction was characterized by complete hind limb paraplegia, loss of muscle tone, and no joint contracture. The dogs moved only with their forelimbs, and the modified canine Basso–Beattie–Bresnahan locomotor rating scale (cBBB) declined from 19 to zero following SCI induction. Furthermore, there was no deep or superficial pain response with an absence of evoked potential conduction across the injured area of the spinal cord as measured by somatosensory evoked potential (SSEP). There were no life-threatening side effects seen across the treatment groups.

Urine and fecal incontinence developed in the dogs shortly after SCI induction. Manual bladder expression was essential on a daily basis until the control group regained voluntary urination and bladder tone at the end of the second week, which recovered slower than the other groups. Six dogs had urinary tract infections following induction (one dog in the control group, three in the MPSS group, and two in the combination group), but none in the calpain inhibitory group. Following SCI induction, one dog in the MPSS group and two dogs in the combination group had signs of gastrointestinal disturbance, which disappeared after one week.

**Figure 2:**
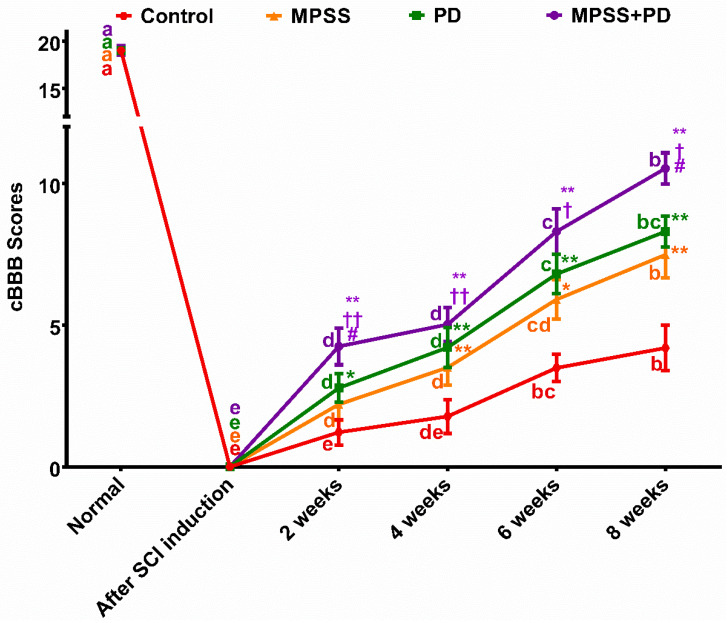
Locomotor scoring of SCI-induced dogs using the modified canine Basso–Beattie–Bresnahan locomotor rating scale (cBBB). Dogs were assessed before SCI induction, on the first day after induction, and subsequently weekly until the end of the study period. Data from different groups were compared to normal preoperative values (baseline). Data were represented as mean ± SD. At the same time point, * denotes significance compared with the control group at *p* < 0.05, ** denotes significance compared with the control group at *p* < 0.01, † denotes significance compared with the MPSS group at *p* < 0.05, †† denotes significance compared with the MPSS group at *p* < 0.01 and # denotes significance compared with the PD group at *p* < 0.05. Meanwhile, within the same group, different letters indicate significant differences *p* < 0.05.

During the course of the investigation, the calpain inhibition, MPSS, and combination groups all demonstrated an improvement in their cBBB score up to an intermediate stage. On the other hand, the control group continued to be in the early stage of cBBB score improvement (Figure 2).

In the second week after SCI induction, it was noted that dogs in the combination group had improved motor function compared to those dogs in the other groups. A modest movement in all of the joints of the hind limb showed this improvement. By the sixth week, three of the dogs in the combination group and one of the dogs in the calpain inhibitor group displayed considerable mobility of all of their hind limb joints with firm placement of their paws on the ground. By the end of the study at eight weeks, two dogs from the MPSS group and one dog from the calpain inhibitory group had symptoms of non-ambulatory paraparesis, a condition in which dogs are unable to bear their own weight and walk. On the other hand, three of the dogs in the combination group and one of the dogs in the calpain inhibitory group exhibited evidence of ambulatory paraparesis, which is a condition in which the dog is able to walk but only very slowly and unsteadily. Unfortunately, the dogs in the control group remained in the early stages of cBBB score improvement (paraplegic). Two dogs in each of the MPSS and calpain inhibition groups remained in the intermediate stage of cBBB score improvement, where the dogs were unable to move the rear limbs but could still feel the rear toes. Meanwhile, one dog in the combination group showed plantar placement of the paw with weight support only when standing.

**Figure 3:**
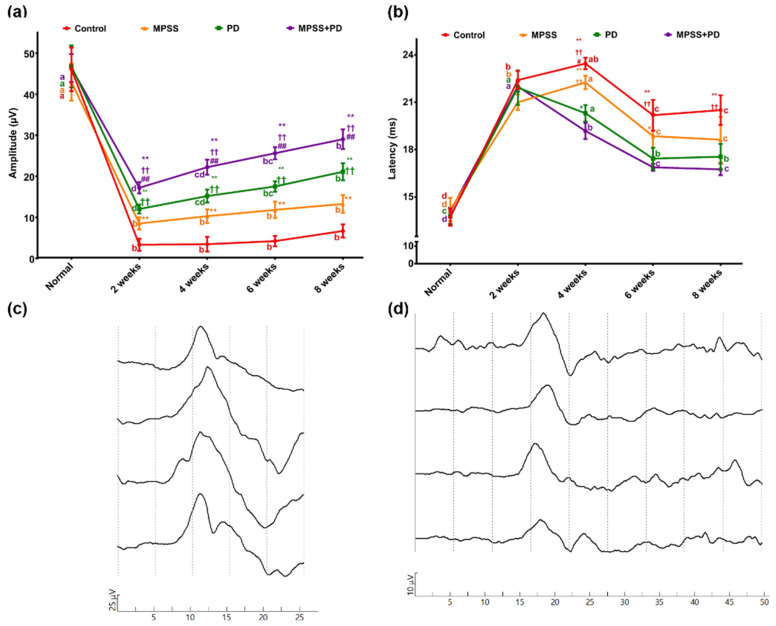
The findings of spinal SSEPs were recorded from sites along the vertebral column after tibial nerve stimulation. (**a**) Quantitative analyses illustrated the changes in baseline-to-peak amplitude (µV) Data were represented as mean ± SD. At the same time point, ** denotes significance compared with the control group at *p* < 0.01, †† denotes significance compared with the MPSS group at *p* < 0.01 and ## denotes significance compared with the PD group at *p* < 0.01. Meanwhile, within the same group, different letters indicate significant differences *p* < 0.05. (**b**) Peak latency (ms) of SSEPs during follow-up in all groups. Data were represented as mean ± SD. At the same time point, * denotes significant latency compared with the combination group at *p* < 0.05, ** denotes significant latency compared with the combination group at *p* < 0.01, †† denotes significant latency compared with the PD group at *p* <0.01 and # denotes significant latency compared with the MPSS group at *p* < 0.05. Meanwhile, within the same group, different letters indicate significant differences *p* < 0.05. (**c**) SSEPs traces were recorded from the combination group before (at baseline) and (**d**) after 8-weeks of induced SCI. Traces were obtained from tibial nerve stimulation. Peak latency (ms) and baseline-to-peak amplitude (µV) measurements were obtained from the first prominent negative peak. Gain = 25 µV/div (**c**) or 10 µV/div (**d**); sweep speed = 5 ms/div (**c**,**d**). Data from different groups were compared to normal preoperative values (baseline).

In all dogs, unilateral spinal SSEPs were recorded from sites along the vertebral column after tibial nerve stimulation. In the control group, the peak latency of the most prominent first negative wave ranged between 13.1 ms and 15.2 ms at baseline and 16.4 ms to 21.5 ms at eight -week follow-up. Baseline-to-peak amplitudes ranged between 36.9 µV and 53.1 µV at baseline and 5.9 µV and 30.4 µV at eight-week follow-up. The combination group displayed significantly lower latency of SSEPs at eight-week in comparison to PD, MPSS, and control groups (16.5 ± 0.4 ms versus 17.6 ± 0.8 ms, 18.6 ± 1.4 ms, and 20.5 ± 0.9 ms, respectively). Additionally, the combination group showed significantly higher amplitude of SSEPs at eight weeks in comparison to PD, MPSS, and control groups (28.9 ± 1.4 µV versus 20.9 ± 1.1 µV, 13.1 ± 2.2 µV, and 6.5 ± 0.6 µV, respectively) (Figure 3). These findings support the notion that combining MPSS with PD150606 therapy improves functional recovery after SCI.

### 2.2. Serum Biochemical Analysis

**Figure 4:**
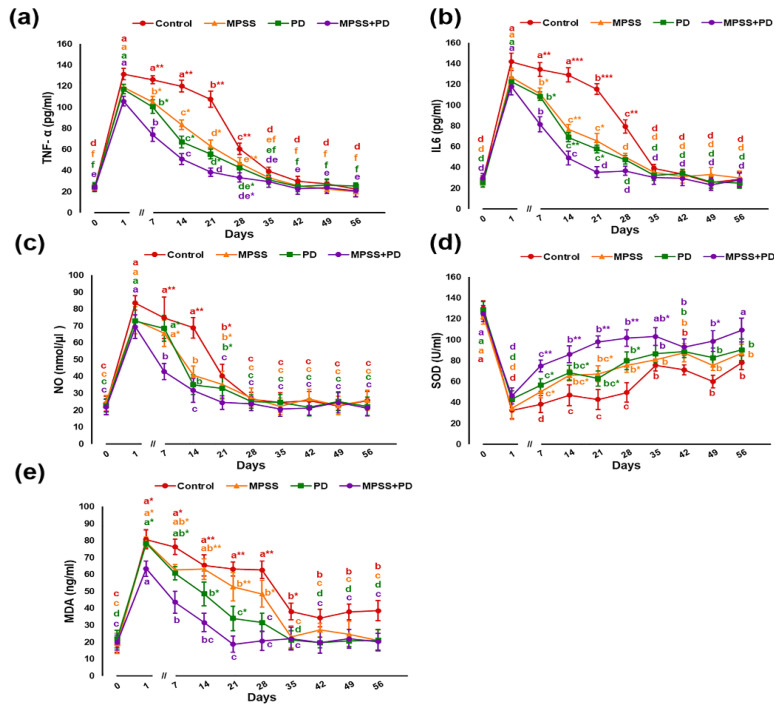
Effect of MPSS, PD, and MPSS+PD treatment on serum levels of pro-inflammatory cytokines and antioxidant status during 8-week study model for SCI. (**a**) Serum TNF-α levels. (**b**) Serum IL-6 levels. (**c**) Serum NO levels. (**d**) Serum SOD activities. (**e**) Serum MDA levels. Data were represented as mean ± SD. Within the same group, different letters indicate a significant difference *p* < 0.05. At the same time point, * indicates a significant difference (*p* < 0.05), ** indicates significant difference (*p* < 0.01) and *** indicates significant difference (*p* < 0.001). Data from different groups were compared to normal preoperative values at 0 day (baseline).

Following SCI, serum concentrations of pro-inflammatory cytokines tumor necrosis factor alpha (TNF-α), interleukin-6 (IL-6), and nitric oxide (NO) demonstrated significant (*p* < 0.05) elevation in all groups (Figure 4a–c). Their levels reach the peak by the first day after injury then slightly reduced by the first week but maintained at higher levels in the control when compared to baseline. The persistence of significant (*p* < 0.05) elevated levels of TNF-α, IL-6, and NO for about three weeks after injury suggests the progression of chronic inflammatory response in injured dogs. Both MPSS and calpain inhibitory groups significantly (*p* < 0.05) counteracted the inflammatory response as indicated by markedly declined levels of inflammatory markers in a time-dependent manner, which started as soon as the first week post injury. Moreover, combining both treatments showed greater efficiency than that produced by either drug alone. Upon examination of biomarkers of antioxidant status and lipid peroxidation (Figure 4d,e), all groups exhibited a significant (*p* < 0.05) decline in serum activity of superoxide dismutase (SOD) with a notable increase in malondialdehyde (MDA) concentration after injury. These changes started on the first day and continued in the control group until the 8th week. However, after the fourth week, changes became less pronounced but remained significant compared to baseline, indicating a probable neurotoxic effect. On the contrary, MPSS and calpain inhibitory groups showed comparable results in improving oxidative stress (OS) associated with SCI. Furthermore, co-administration of MPSS and PD offered a superior method for controlling SCI-induced OS.

### 2.3. Histopathological Evaluation of the Therapeutic Efficacy of MPSS and the Calpain Inhibitor PD150606 following SCI

**Figure 5:**
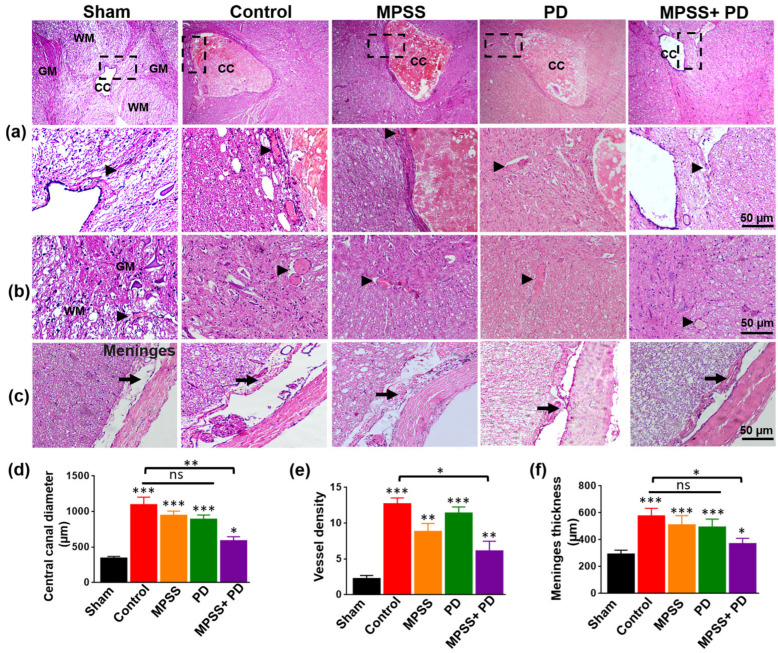
Histopathological findings of control, MPSS, PD150606, and MPSS+PD150606 groups 8 weeks post SCI. (**a**–**c**) Representative microscopy images of the dog’s spinal cord in the indicated groups after experimentally induced SCI, stained with H&E. (**a**) Gay matter (GM), white matter (WM) and the central canal (CC) of the spinal cord are shown in low and high magnification (rectangular). (**b**) Neuronal cell bodies and glial cells from the ventral horn, and (**c**) spinal cord meninges are shown. Compared to the sham group, all groups showed severe spinal canal congestion, hemorrhage with many congested blood capillaries (arrows heads), infiltration of microglial cells, neuronal loss and damage, and an apparent increase in spinal meninges thickness (arrows). However, the combination group of MPSS and PD150606 revealed less hemorrhaging, fewer inflammatory responses, and congested blood capillaries compared with other groups. Scale bare = 50 µm. (**d**) Quantitative analysis of the average central canal diameter, (**e**) Vessel density (vessels/0.5 mm^2^), and (**f**) meninges thickness, in indicated groups. Ten images from each group were used for data quantification. Data are presented as the mean ± SD. * indicates a significant difference (*p* < 0.05), ** indicates a significant difference (*p* < 0.01), *** indicates a significant difference (*p* < 0.001), and ns indicates non-significant.

The injured spinal cord showed severe spinal canal congestion, hemorrhage with numerous congested blood capillaries, infiltration of microglial cells, neuronal loss, and damage compared to the intact spinal cord (Figure 5a,b). Additionally, the meningeal thickness in the spinal cord increased markedly (Figure 5c). Compared to other groups, the combined group showed less hemorrhaging, fewer inflammatory responses, slightly congested blood capillaries, and a small increase in spinal meninges (Figure 5a–c). The combination group had significantly lower percentages of the congested central canal, vessel density, and meningeal thickness compared to other groups treated with only MPSS or PD150606 (Figure 5d–f).

**Figure 6:**
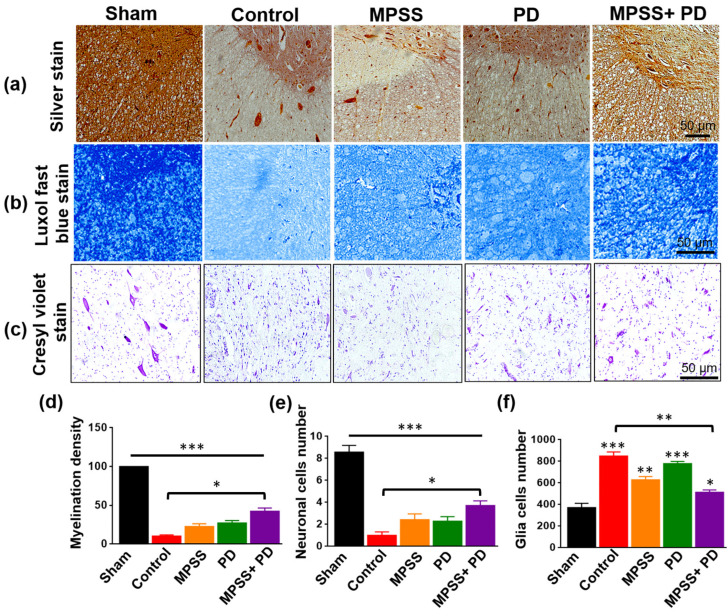
The combination groups of MPSS+PD150606 preserve the axons’ myelination after SCI. (**a**) Histological analysis of spinal cord lesions stained with Bielschowsky’s silver, and (**b**) Luxol fast blue for myelination, and (**c**) Cresyl violet stain for neuronal cell bodies and Nissl granules. All groups exhibited severe demyelination of nerve fibers, neuronal loss, and gliosis in the SCI lesion. The myelination density, neuronal cells, and glia cells were dramatically preserved in the combination group compared to other groups. Scale bare = 50 µm. (**d**) Quantitative analysis of the average myelination density, (**e**) neuronal cell number (cells/0.1 mm^2^), and (**f**) Glial cells number (cells/0.1 mm^2^) in the indicated groups. Ten images from each group were used for data quantification. Data are presented as the mean ± SD. * *p* < 0.05, ** *p* < 0.01, *** *p* < 0.001, and ns indicates non-significant.

All groups had extensive demyelination of nerve fibers, as shown by Bielschowsky’s silver and Luxol fast blue staining. However, in the combination group, myelination appeared to be preserved to a greater extent (Figure 6a,b,d). Furthermore, all groups demonstrated neuronal cell damage and an increase in the number of glial cells. However, in the combination group, neuronal loss and gliosis were somewhat reduced (Figure 6c,e,f). These data imply that combining MPSS with PD150606 treatment preserves neuronal integrity and axon myelination after SCI.

### 2.4. Assessment of Glial Inflammatory Markers and Calcium Binding Proteins

**Figure 7:**
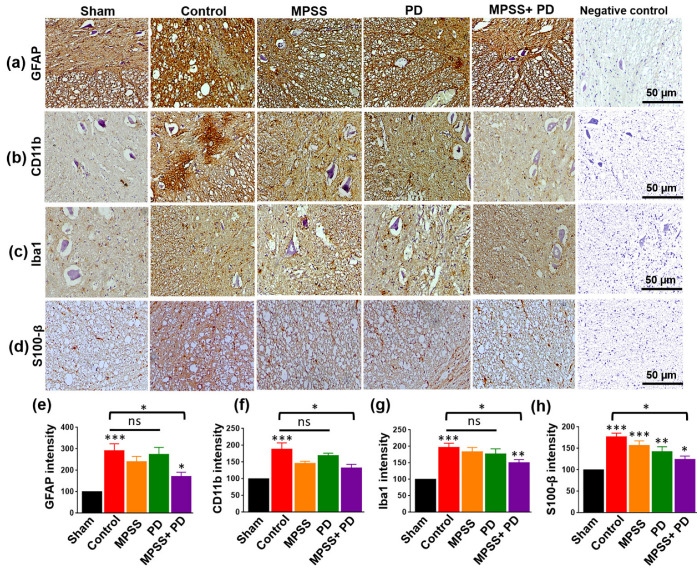
Immunohistochemical assessments of glia inflammatory markers and calcium binding proteins following different treatments after SCI. (**a**) Representative images immunoassayed with glial fibrillary acidic protein (GFAP), and (**b**) CD11b of activated astrocytes and microglia, receptively, of the spinal cord from indicated groups. Strong staining of GFAP and CD11b positive cells was found in the spinal cord lesion of the control group, MPSS and PD150606, while the combination group had a remarkably better inhibitory effect on glia cells activation. Similar results were obtained when (**c**) sections of the spinal cord were stained with the calcium-binding proteins Iba1, and (**d**) S100-β in glial cells. The calcium-binding proteins were increased in all groups; but the combination group remarkably inhibited their upregulation compared to other groups. Scale bare = 50 µm. Negative control for antibody reaction was used where non-specific secondary antibody was used. (**e**) Quantitative analysis of the average GFAP intensity, (**f**) CD11b intensity, (**g**) Iba1 intensity, and (**h**) S100-β intensity in the indicated groups. Ten images from each group were used for data quantification. Data are presented as the mean ± SD. * *p* < 0.05, ** *p* < 0.01, *** *p* < 0.001, and ns indicates non-significant.

SCI is accompanied by an increased and long-lasting inflammatory response [26,27,28]. Consequently, the expression of the astrocyte-specific marker glial fibrillary acidic protein (GFAP) and the microglial inflammatory marker CD11b was examined following SCI. The spinal cord lesion showed a remarkable elevation in GFAP and CD11b immunostaining compared to the sham group. In contrast, GFAP and CD11b levels were lower in the combination group. However, their levels were not statistically different in MPSS or PD alone from the sham group (Figure 7a,b,e,f). Similarly, the combination reduced the elevated levels of ionized calcium binding adaptor molecule 1 (Iba1) and S100 calcium-binding protein B (S100-β) following SCI (Figure 7c,d,g,h). Of note, MPSS and a calpain inhibitor minimize the activation of astrocytes and microglia.

### 2.5. Inhibition of Calpain Abolishes the Generation of p25 and, Consequently, the CDK5 Activation in SCI

**Figure 8:**
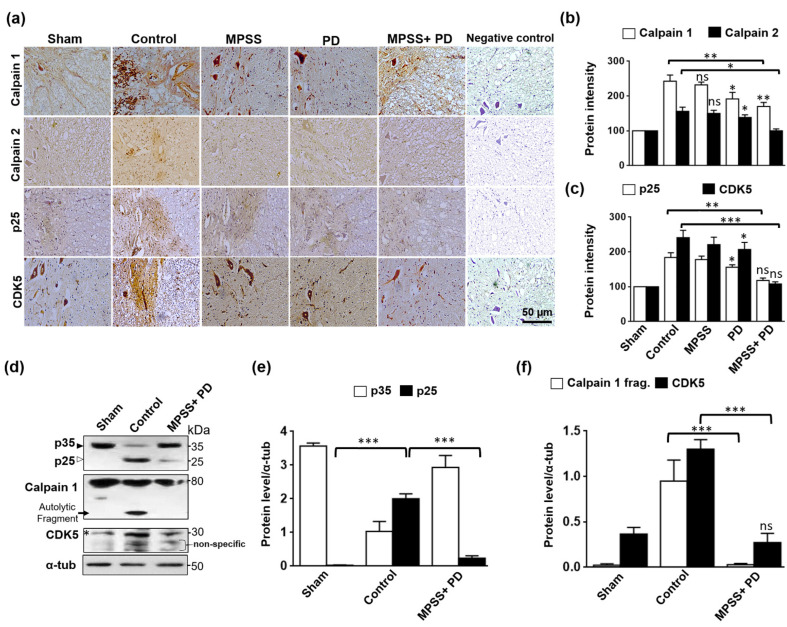
Inhibition of calpain abolishes the generation of p25 and consequently the CDK5 activation. (**a**) Representative images immunoassayed with calpain1, calpain 2, p25, and CDK5 antibodies from the indicated groups. The increased expression of the indicated proteins was not significantly reduced in the MPSS group, but was significantly attenuated in PD150606 and the combination group. Negative control for antibody reaction was used where non-specific secondary antibody was used. (**b**) Quantitative analysis of the average protein intensity of both calpains 1 and 2, while (**c**) intensity of p25 and CDK5 in the indicated groups. Ten images from each group were used for data quantification. Data are presented as the mean ± SD. * *p* < 0.05 and ** *p* < 0.01. (**d**) Immunoblots show proteolytic cleavage of p35 to p25, autolytic fragments of calpain, and CDK5 protein levels in spinal cord lysates from the indicated groups. There was no p35 cleavage and autolytic calpains fragment in the sham group. Truncation p35 to p25 was increased in the spinal cord injury of the control group following SCI. This truncation was remarkably inhibited in the combination group of MPSS+PD150606. The black arrowhead indicates the full length of p35 protein, while the white arrowhead indicates the truncated p25 protein. Black arrows indicate the autolytic calpain 1 fragment upon calpain activation. Star indicated the specific band of CDK5 at 30 kDa. α-tubulin was used as a loading control. (**e**) Bar graphs show relative levels of the p35 and p25, and (**f**) calpain 1 fragments and CDK5 protein densities in indicated groups. Data are presented as the mean ± SD (*n* = 3). *** *p* < 0.001, and ns indicates non-significant.

Micro (μ)-calpain and mili (m)-calpain (or calpain 1 and 2, respectively) are two distinct calpains that differ primarily in their in vitro calcium (Ca^2+^) requirements [17,18]. Calpain activation triggered by Ca^2+^ influx caused by disease or injury is a well-known observation in the brain and spinal cord pathology [22,29]. As expected, following SCI, specific intense staining of calpain1 and calpain 2 was found in the spinal cord lesion (Figure 8a). This increase in both calpains was partially decreased by the calpain inhibitor PD150606 alone, but not by MPSS. However, the combination of MPSS and PD therapy dramatically lowered calpain levels after SCI (Figure 8a,b).

Calpain activation triggers p35 to p25 cleavage, resulting in the persistent activation and mislocalization of cdk5, which leads to neuronal death [30]. To preliminary examine the mechanism of triggered neuronal cell death, p25 and cdk5 were immunoassayed in spinal cord lesions. The results show that the expression of p25 and cdk5 was markedly increased in the control group, but this increase was significantly inhibited in different treatments, with therapy with PD150606 alone or PD150606 combined with MPPS being better than treatment with only PD150606 (Figure 8a,c).

Further immunoblotting analysis was used to investigate the molecular mechanism of both the PD150606+MPSS therapy and the Cdk5 activation. As hypothesized, calpains cleaved p35 to 25 kDa fragments in the control group, but this cleavage was inhibited in the PD150606 and MPSS combination group (Figure 8d,e). In addition, the increased calpain fragments, which showed their activation after SCI, were apparently abolished by PD150606 and MPSS therapy (Figure 8d,f). As a result of using both PD150606 and MPSS, the total protein levels of Cdk5 were reduced to normal levels when compared to the control group (Figure 8d,f).

### 2.6. Inhibition of Calpains Abolishes the Increase in Tau Hyperphosphorylation after SCI

**Figure 9:**
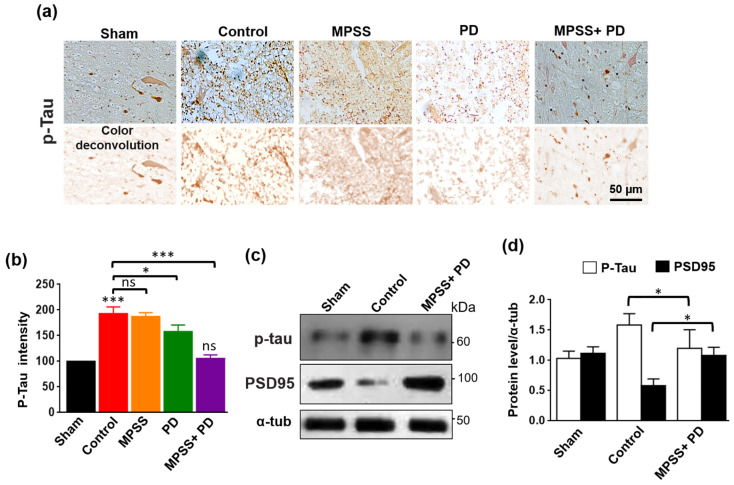
Inhibition of calpain attenuates tau hyperphosphorylation after SCI. (**a**) Representative images of phosphorylated-tau (AT8)-stained sections of the spinal cord from indicated groups after SCI. Specific staining of AT8 was upregulated in the control group, while MPSS+PD150606 administration prevented this activation. (**b**) Quantitative analysis of the average p-tau intensity. Ten images from each group were used for data quantification. Data are presented as the mean ± SD. * means *p* < 0.05, *** *p* < 0.001, and ns indicates non-significant. (**c**) Immunoblots showed the total protein levels of phosphorylated-tau and the scaffold postsynaptic protein PSD95 in the indicated groups. The treatment with MPSS+PD150606 abolished the increased levels of p-tau while preserving the total protein levels of PSD95 after SCI. (**d**) Bar graph shows relative protein levels of p-tau and PSD95 in the indicated groups. Data are presented as the mean ± SD (*n* = 3). * means *p* < 0.05.

**Figure 10:**
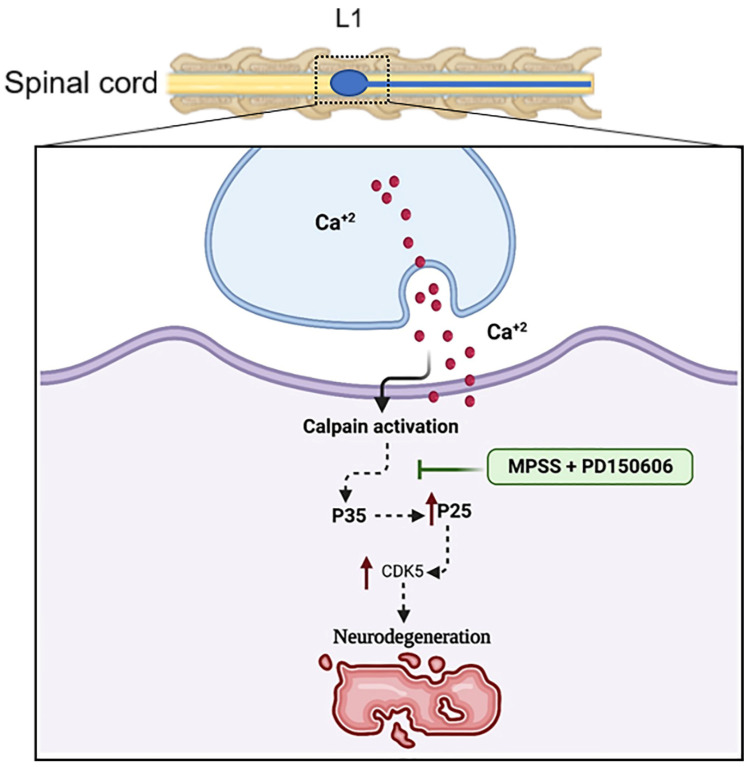
The calpain inhibition mechanism in SCI. Spinal cord compression causes an increase in calcium influx. The cleavage of p35 to p25 caused by calcium-dependent calpain activation leads to Cdk5 activation, which causes neurodegeneration. The MPSS and PD150606 combination inhibit calpain activation to maintain neuronal integrity.

The complex Cdk5-p25 is involved in neuronal cell death and tau hyperphosphorylation [31]. Tau proteins are microtubule-associated proteins, which are known to maintain the integrity and polarity of neuronal and glial microtubules. Tau protein phosphorylation is believed to play a crucial part in the pathogenesis of tauopathies and neurodegenerative disorders [21,32]. After SCI, immunostaining and immunoblotting analysis revealed that phosphorylated tau (AT8) expression was upregulated in the control group, but MPSS and PD therapy significantly reduced this upregulation (Figure 9a–d). While this combination treatment maintained the total protein levels of PSD95 to normal levels after SCI (Figure 9c,d). These findings suggest that calpain inhibition could be able to prevent excessive calpain activation and tau hyperphosphorylation in SCI. The working theory beyond the role of calpain inhibition and MPSS after SCI is elucidated in (Figure 10).

## 3. Discussion

This study aims to assess the therapeutic benefits of IV-administered calpain inhibitor PD150606 in a canine SCI model, as well as to combine PD150606 with MPSS, which is employed as a neuroprotective drug in the treatment of acute SCI [33]. According to the current findings, combining MPSS with a calpain inhibitor had a better impact in preventing calpain-mediated neurodegeneration after SCI than using either MPSS or PD150606 treatment alone.

The development of a model of irreversible acute SCI is crucial. Dog paraplegia has served as a valuable model for developing novel human therapies for SCI. According to several reports, dogs are a useful transitional biological model, spanning the huge gap between human and rat investigations [12,14,34].

The findings of this study showed that canine paraplegic models could be created safely utilizing a minimally invasive balloon compression method, resulting in injuries comparable to intervertebral disk herniation in dogs and humans [9,35,36]. Furthermore, no difficulties were identified in this investigation regarding the induction approach, which is entirely percutaneous, as compared to Fukuda’s SCI model [37]. The balloon-induced SCI strategy was chosen over other induction techniques because it is a straightforward procedure that causes no damage to the surrounding tissues, the dosage response depends on balloon volume, and the degree of injury occurs in dogs [38], in contrast to the previous Purdy’s models [39,40]. Embolectomy catheters were selected because they are stiffer and less irritating than other Forgaty catheters [41].

Dogs in this study showed locomotor improvement to varying degrees in different groups, manifested by enhanced range of joint motion and occasional weight bearing of the hind limb. These findings were supported by SSEP findings that revealed increased amplitude and decreased latency in the combination group by the end of the study compared to the PD or MPSS group alone. In this study, dogs suffered from gastrointestinal disturbance following treatment with MPSS. This could be attributed to the fact that the administration of glucocorticoid steroids causes many complications, including gastrointestinal problems and an increased incidence of infection [16,42].

Following SCI, the dogs in the study had cBBB scores of less than one. The SSEP findings demonstrated a substantial decrease in wave amplitude and an increase in wave latency and duration time in all groups. By the end of the study period, dogs in various groups exhibited locomotor improvement ranging from slight movement of all hind limb joints in the control group to consistent weight bearing of the hind limb in the combination group (eight weeks). The difference in cBBB scores between the combination group and the other groups was significant (*p* < 0.05). The improvement in locomotion overtime was in line with the SSEP results of decreasing wave latency with increasing amplitude.

The increased neuronal damage in SCI is linked to calpain activation, as shown in the present work and other previously reported investigations [17,18,43,44]. Therefore, identifying the cellular and molecular pathophysiological mechanisms that cause spinal cord damage can contribute to the development of novel treatment strategies [4,18]. The primary injury induces rapid cell death or necrosis, followed by a secondary subsequent spinal cord pathology that is caused mainly by calpain activation, cell death, inflammatory response, OS [45], lipid peroxidation [46], neuronal excitotoxicity [47], and excess cytokine release at the site of the original injury [48].

The extent of secondary neurodegeneration following SCI is thought to depend on the severity of the inflammatory processes. Neuroinflammation is initiated by activating monocyte-derived macrophages and resident microglia at the site of injury, releasing robust amounts of pro-inflammatory cytokines, including TNF-α, IL-1, and IL-6, along with uncontrolled production of inducible nitric oxide synthase, resulting in excessive production of NO [4,8]. This study’s MPSS+PD150606 combination therapy preserved neuronal integrity and axon myelination following SCI. MPSS was shown to mitigate secondary damage by reducing inflammation, spinal cord ischemia, and inhibiting cell membrane lipid peroxidation [49,50].

Ischemic insults result in an elevation of intracellular Ca^2+^ [44], with subsequent activation of calpains. Ca^2+^ and glutamate overload are neurotoxic since they can trigger prolonged excitatory synaptic transmission, a condition known as neuronal excitotoxicity [51]. Therefore, calpain inhibition has been shown in some trials to be neuroprotective [22,52,53]. Consequently, medications interfering with one or more of these pathways could reduce the occurrence of SCI.

PD150606 is a cysteine protease inhibitor that inhibits calpains and suppresses calpain-mediated cell death, leading to reduced neuron loss and abnormal calpain activation [54]. In this study, after SCI, there was a significant increase in calpain activity evident by immunostaining and western blotting in the injured spinal cord. However, MPSS and PD150606 combination therapy showed preservation of the neuronal integrity and axon myelination. This could be attributed to MPSS inhibiting calpain in vitro [49,50]. Reactive astrocytes intertwine their processes in the immediate location of damage to generate an anisomorphic gliosis barrier [4,27]. In this study, GFAP, and microglia protein CD11b expression were increased in reactive astrocytes following SCI. The use of a combination of MPSS and a calpain inhibitor to treat SCI minimizes the activation of astrocytes and microglia.

Herein, results indicated a chronic inflammatory response driven by peak pro-inflammatory cytokine production for a prolonged time in the control group [55]. Even the observed reduction in inflammatory mediators by the fourth week in control dogs does not necessarily indicate a reduced body response because delayed TNF-α and IL-6 inhibition is rendered ineffective due to the progression of chronic inflammation in the injured spinal cord [56]. This could promote a sequence of malicious events in nerve cells, resulting in neuronal degeneration and dysfunction [57]. The data of the current study are consistent with those obtained by Yu and Qian [58], Spitzbarth et al. [59], and Pusterla et al. [60], who revealed the production of large amounts of pro-inflammatory mediators following SCI in rats, dogs, and horses, respectively.

Neurons of the central nervous system are sensitive to oxidative damage by OS due to a high amount of polyunsaturated fatty acids [61] with relatively low antioxidant capability [62]. This explains the depletion of antioxidant enzyme activity, whereas elevated MDA levels in the control group. Similar to our results, Naik et al. [63], Morsy et al. [64], Marquis et al. [65], and Fu et al. [66] reported reduced antioxidant enzyme systems with increased biomarkers for OS and lipid peroxidation in different models of neurological injuries.

Treatment of injured dogs after SCI with MPSS or PD reduced the inflammatory response, retrieved antioxidant enzyme equilibrium and diminished lipid peroxidation. This is manifested by the significant suppression of NO, TNF-α, IL-6, and MDA levels in contrast to elevated SOD activity. In line with the findings of this study, several reports recorded significant declines in TNF-α, IL-6 [67,68], and NO [69] with an enhanced antioxidant capacity [70,71] upon treating acute SCI with MPSS. Furthermore, the PD results agreed with previous studies by Liu et al. [72], Ni et al. [73], and Chen et al. [74]. Furthermore, these data suggested that PD might display better neuroprotective potential when compared with MPSS. This could be attributed to being a specific calpain inhibitor and hence possessing the advantage of controlling an extra neurotoxic pathway, which will eventually enhance recovery of SCI.

Cdk5 is a kinase that phosphorylates tau protein and is regulated by its endogenous activators, p35 and p25 [22,30]. Calpain was identified to cleave p35 into p25, a truncated protein with more kinase activity than p35. However, the mechanism of p35-p25-Cdk5 activation and tau hyperphosphorylation in SCI is unclear. Immunohistochemistry and immunoblotting revealed upregulation of p35 and its truncated product p25 expressions in the spinal cord lesion and its penumbra in the current investigation. The neuroprotective impact of MPSS and PD150606 in SCI might be mediated through suppression of p35-p25-Cdk5 activation. Most tau research focuses on neurodegenerative Alzheimer’s disease [21], and there are many studies of tau hyperphosphorylation in SCI models [22,31,32]. Distinct epitopes are present in the phosphorylation-dependent anti-tau antibodies. AT8 only interacts with tau when Ser199, Ser202, and Thr205 are all phosphorylated in close proximity to one another. AT8 reactivity cannot be achieved with a single phosphorylation of any of the residues [22]. Therefore, for protein-directed kinases, such as Cdk5 complexes, AT8 is helpful and highly specific in identifying Ser202/Thr205 phosphorylation. The current study’s findings demonstrated that implementation of calpain inhibition therapy after SCI might prevent abnormal tau hyperphosphorylation following SCI.

Although the calpain inhibitor PD has shown effectiveness in in vitro studies in a cultured cell model, the development of this inhibitor in clinical trials has been hampered mostly by a lack of calpain isoform specificity and the general reactivity of the inhibitors in the animal model. Furthermore, differences in the chemical components, mechanisms of action, or specificity of calpain inhibitors may be responsible for variations in the degree of calpain level inhibition in vivo [18]. It should be noted that previous research showed that SCI significantly increases intracellular free calcium levels even though SCI needs strong selective and specific inhibitors at the proper dose and therapy duration [44]. For the first time, we demonstrated in this work the potential for using PD in a clinical trial for the treatment of SCI. The impact of PD on calpain activation was slight, yet it was substantial when compared to control. Additionally, the combination of E-64-d and MP was more effective than either E-64-d or MPSS alone in inhibiting calpain levels in SCI in rats [53].

Finally, the current findings suggest that early calpain inhibition following acute SCI by administration of both MPSS and PD150606 may prevent further neurodegeneration by inhibiting p35/p25-Cdk5 activation. Calpain inhibition protects against the progression of spinal cord damage via targeting the main molecules incorporated in inflammation and neurodegenerative pathways. Additionally, this therapy could be employed as an alternative therapeutic approach for acute SCI

## 4. Materials and Methods

### 4.1. Study Design

The study was carried out at the Department of Surgery, Anesthesiology and Radiology, Faculty of Veterinary Medicine, Suez Canal University. G*Power version 3.1.9.2 [75] was used to calculate the sample size to estimate the minimum number of dogs. The effect size f was 1.10 using alpha (α) level of 0.05 and Beta (β) level of 0.05, i.e., power = 80%; the estimated sample size (*n*) should be at least 16 dogs. Based on the sample size calculation, seventeen healthy mongrel male dogs aged 12–36 months (23.06 ± 7.5) and weighing 19–24 kg (20.09 ± 1.42) were divided into four groups: Control group (saline was given after SCI induction; *n* = 4); MPSS group (MPSS was given after SCI induction; *n* = 4); calpain inhibitor group (PD150606 was given after SCI induction; *n* = 4); and the combination group (both MPSS+PD150606 were given after SCI induction; *n* = 4). Furthermore, one dog participated in a sham group (without SCI induction). The dogs were fed standard food that met the National Research Council’s (NRC) nutritional standards for dogs [76] and had free access to water. Dogs were maintained at the animal house for two weeks prior to the experiment for acclimatization and a thorough inspection of any health concerns discovered during repeated physical examinations or abnormal neurological disorders. During the acclimatization period, dogs’ serum biochemical parameters and total blood count were all within normal ranges. Exclusion criteria included any health or neurological status problems.

### 4.2. Induction of SCI

Prior to SCI induction, the dogs were fasted for 12 and 2 h for food and water, respectively. The dogs were given intramuscular (IM) injections of chlorpromazine hydrochloride (Neurazine, Misr Co. Pharm., Cairo, Egypt) at a dose of 1 mg/kg, nalbuphine HCl (Nalufin^®®^, Amoun Pharmaceutical Company, Obour City, Egypt) at a dose of 1 mg/kg, and atropine sulfate (Memphis Pharmaceutical, Cairo, Egypt) at a dose of (0.04 mg/kg). After that, general anesthesia was induced via intravenous (IV) injection of Propofol (Diprivan^®^, AstraZeneca, Cambridge, UK) at a dose of (2 mg/kg) and maintained via 2% isoflurane (IsoFlo^®^, Zoetis, Kalamazoo, MI, USA) and oxygen [77].

The lumbosacral area was clipped, shaved, and aseptically prepared. A minimally invasive balloon compression approach was used for SCI induction [36]. Radiography was taken to measure and validate the length between the lumbosacral space and the first lumbar segment (L1) vertebral body in each dog before operation. The lumbosacral space was detected after the dogs were placed in the recumbent position. A 22-gauge spinal needle was introduced via the lumbosacral space. Aspiration was done to confirm the absence of blood and cerebrospinal fluid. A guide wire was then inserted via the spinal needle till lumber vertebra No.1 (L1), and the spinal needle was withdrawn after the guide wire was located at the L1. A 5Fr introducer and dilator (Check Flo Performer^®^ introducer set, Cook Medical, Bloomington, IN, USA) were inserted into the epidural space using the guide wire, and the dilator and guide wire were then removed, leaving the introducer only. After that, a 5F emblectomy catheter (ISO MED, ZAE, Chambly, France) was introduced via the introducer into the epidural space and advanced into the cranial direction until it reached the cranial margin of the L1 vertebral body, to a distance corresponding to the distance previously measured between the lumbosacral space and L1. The site was confirmed using radiography and then the balloon was inflated by injecting iohexol (Omnipaque, GE Healthcare) at a dose of 50 µL/kg diluted with saline in a 50:50 proportion until it reached the desired final volume in the epidural space. Plain radiography and CT were performed to confirm the position and shape of the balloon. The balloon was fixed with a Chinese finger-type suture. The catheter was deflated and removed after 12 h [78].

### 4.3. Postoperative Management and Administration of MPSS and Calpain Inhibitor

Dogs were caged separately under close observation for general health status as well as food and water intake. All dogs were injected IM with cefotaxime (Cefotax, EIPICO, Tenth of Ramadan, Egypt) at a dose of (50 mg/kg, every 8 h for 5 days). Tramadol (Amriya for Pharmaceutical Industries S.A.E) was injected IV at a dose of (4 mg/kg, every 8 h for 5 days) for pain management. Manual bladder expression was performed every 6 to 8 h until spontaneous urine control was restored. The frequency of evacuation was determined by the extent of the previous evacuation and the amount of urine produced [79]. Soft pads were provided to prevent pressure sores. In the MPSS group, MPSS (SOLU-MEDROL^™^; Pfizer, NY, USA) was injected IV at dose of (30 mg/kg, every 6 h for 2 days) [34]. In the calpain inhibitor group, dogs were injected IV with PD150606 (ab141464, Abcam, Cambridge, UK) dissolved in 1% dimethyl sulfoxide (DMSO) at a dose of (1.0 mg/kg every day for 21 days) [20]. In the combination group, both MPSS and PD150606 were injected as previously described.

### 4.4. Postoperative Clinical Follow-Up

Neurologic examination and gait assessment were performed on the dogs 24 h after induction of SCI till the end of the study (8 weeks) to evaluate functional recovery as described by David et al. [80]. Two blinded investigators evaluated and graded each dog’s gait and performed neurological tests on them. The evaluation involved determining if the dog’s gait is ambulatory paraparetic, nonambulatory paraparetic, or paraplegic. Locomotor scoring of SCI-induced dogs was carried out using cBBB before and 24 h after SCI induction and subsequently at 2, 4, 6, and 8 weeks, as previously described by Song et al. [12]. Moreover, the dogs were put in lateral recumbency and assessed for the presence of superficial and deep pain, as well as conscious proprioception while supported upright. During testing for superficial pain responses, skin on the flank and limbs was squeezed severely using a hemostat. A severe pinching of the paw’s medial and lateral digits indicated the deep pain response. For comparison, positive results were obtained by assessing the forelimbs [14].

### 4.5. Serum Biochemical Analysis

The MDA level was determined using a commercially available kit to determine the level of lipid peroxidation [81] (Cat. No. LS-F28018, Lifespan Biosciences Co., Seattle, WA, USA). Measurement of NO levels was evaluated by detecting its stable metabolites, nitrite and nitrate, as previously described by Miranda et al. [82] (Cat. No. MBS2604161, MyBioSource Co., San Diego, CA, USA). Using a commercially available kit, the activity of the SOD enzyme in the serum was determined following the manufacturer’s instructions [83] (Cat. No. MBS036924, Elabscience Co., Houston, TX, USA). Serum inflammatory markers, including TNF-α, and IL-6 were quantified using ELISA assay kits according to manufacture instruction (Cat. No. MBS025858 and MBS2606513, MyBioSource Co., San Diego, CA, USA), respectively [84].

### 4.6. Somatosensory Evoked Potential (SSEP)

Using standard methods, SSEPs were recorded from the vertebral column [85]. The tibial nerve immediately proximal to the tarsal joint was stimulated individually with a surface electrode. A sufficient pulse intensity (mA) was produced to induce a minimally perceptible movement (contraction) in the distal limb (i.e., just above the motor threshold). Continuous, repetitive, rectangular stimuli of 0.1–0.2 ms pulse duration were applied to the nerve at a stimulus rate (frequency) of 3.0–4.0 Hz. For spinal SSEP recordings, the reference monopolar electrode was put in the epaxial muscle 1 cm lateral to the recording electrode, which was positioned on the vertebral lamina ipsilateral to the tibial nerve stimulation. Recordings were commenced at the cranial aspect of the lumbar vertebra. The baseline-to-peak amplitude and peak latency of the potential were recorded for analysis. Filters (10 to 4000 Hz) were employed to avoid activity unrelated to the generator under study that would interfere with the recordings. Signal averaging was applied to differentiate signals of interest from other interferences. The Neuro-soft 2 Channels NEURO-MEP-MICRO device (NEUROSOFT Russia^®^) was used to perform the electrophysiological evaluation.

### 4.7. Histopathological and Immunohistochemical (IHC) Analysis

By the end of the study (8 weeks), dogs were euthanized with an IV overdose of sodium pentobarbital [86]. The spinal cord samples were immediately preserved and fixed in buffered neutral formalin at a concentration of 10%. Sections were processed histologically in accordance with Layton et al. [87]. For general histopathological examination, histological slices (7 µm thick) were stained with hematoxylin and eosin stain (H&E). Modified Bielschowsky’s silver stain [88] and Luxol fast blue stain [89] were used for the detection of spinal cord myelination. The Cresyl violet stain was used to stain the neuronal cells and Nissl granules. The number of microvessels per unit area was used to indicate vascular density (0.2 mm^2^).

On dewaxed paraffin sections, the IHC method was adjusted as previously described by Buchwalow and Böcker [90]. The primary antibodies used in this study: Goat-polyclonal anti-GFAP (1:200, Cat. No. sc-6170, Santa Cruz, Dallas, TX, USA), mouse-monoclonal anti-CD11b (1:250, Cat. No. 634513, clone OX-42, Millipore, MA, USA), rabbit-polyclonal anti- Iba1 (1:300, Cat. No. 019–19741, Wako, Osaka, Japan), rabbit polyclonal anti-S100-β (1:200, Cat. No. ab41548, Abcam, Cambridge, UK), rabbit polyclonal-anti-calpain 1(1:200, Cat. No. ab39170, Abcam, Cambridge, UK), mouse anti-calpain 2 (1:100, Cat. No. C268, Sigma-Aldrich, St. Louis, MO, USA), rabbit-monoclonal anti-p35/p25 (1:100, Cat. No. C64B10, Cell Signaling), rabbit monoclonal anti-CDK5 (1:250, Cat. No. ab40773, Abcam, Cambridge, UK) and mouse-monoclonal anti-p-tau (AT8) (1:100, Cat. No. MN-1020, Thermo Fisher, MA, USA). The horseradish peroxidase (HRP) conjugated secondary antibodies used in this study included HRP-conjugated anti-goat IgG (1:1000, Cat. No. A9044, Sigma-Aldrich), HRP-conjugated anti-rabbit IgG (1:1000, Cat. No. A0545, Sigma-Aldrich) and HRP-conjugated anti-mouse IgG (1:1000, Cat. No. A9044, Sigma). For the final detection of the colorimetric reaction, 3,3’-diaminobenzidine (DAB) solution was applied as a chromogen. Image J was used to quantify the intensities of immunostaining [91]. Nuclear staining was used as an internal control. An arbitrary thresh-old value was determined for each channel, and it was applied to all relevant images. The average intensity of each protein signal within the region of interest (500 µm^2^) was normalized to the average intensity of the control signal. Non-specific secondary antibodies were used in negative control experiments. The software Image J’s color deconvolution plugin was used to measure the intensity.

### 4.8. Western Blotting Analysis

The frozen spinal cord samples were used for western blot analysis in accordance with the previously published protocol [16]. Briefly, the tissue was homogenized with a sonicator in RIPA lysis buffer (50 mM Tris-HCl at pH 7.4, 150 mM NaCl, 0.1% SDS, 1% NP-40) containing 1X protease inhibitors (Set 1, Roche) on ice for 30–40 min. Lysates were centrifuged (10 min at 13,000 rpm, 4 °C), and protein concentrations were determined using the Bradford method. Equal amounts of protein (15 μg) were resolved by electrophoresis on 10% sodium dodecyl sulfate–polyacrylamide (SDS) gels and transferred to polyvinylidene fluoride (PVDF) membranes. After blocking with 5% skimmed milk for 1 h, the membranes were washed with Tris-Buffered Saline Tween-20 TBST (10 mM Tris–HCl, pH 7.6, 150 mM NaCl, 0.05% Tween-20). The membrane blots were incubated with the following antibodies: anti-calpain 1(1:1000), anti-p35/p25 (1:2000), rabbit monoclonal anti-CDK5 (1:2000), anti-p-tau (1:1000), rabbit polyclonal anti-PSD95 (1:1000, Cat. No. ab18258, Abcam), and mouse anti-α-tubulin (1:20,000, Cat. No. D50297, Millipore). After washing the membrane in TBST, the primary antibodies were detected using goat anti-rabbit IgG or goat anti-mouse IgG conjugated to HRP (1:25,000). Bands were visualized using a chemiluminescent HRP substrate (Millipore). Image J was used to quantify the positive signals on western blots from multiple independent repeats and they were normalized to the loading control to determine the protein levels of the target proteins. The ratio of the amount of calpain autolytic fragment density to a predefined reference measurement using Image J was shown using an arbitrary unit.

### 4.9. Imaging

An Olympus BX41 research optical microscope and an Olympus DP25 digital camera (Tokyo, Japan) were used to capture all H&E and immunoassayed sections. The same settings were used in all images.

### 4.10. Statistical Analysis

All data were collected, calculated, tabulated and statistically analyzed using the following statistical tests. A normality test (Kolmogorov–Smirnov) was performed to check the normal distribution of the samples. Descriptive statistics was calculated in the form of Mean ± Standard deviation (SD). One-way ANOVAs was used to compare between groups at each time point. In some experiments, the statistical significance was determined using paired sample *t*-tests (*p* < 0.05). For non-parametric variables, the Kruskal–Wallis test was performed. Tukey’s post hoc test was performed for the evaluation of statistical significance among the groups. *p* values of <0.05 were considered statistically significant. All statistical analyses were performed using the computer program SPSS for Windows version 26.0 (Statistical Package for Social Science, Armonk, NY, USA: IBM Corp). The graphs were created using GraphPad Prism software version 6 (GraphPad Software, Inc., La Jolla, CA, USA).

## Data Availability

The data that support the finding of this study are available from the corresponding authors upon reasonable request.

## References

[1] Min J., Kim J.Y., Seo C.H., Jeon S.R., Choi K.H., Jeong J.H. (2014). Changes of the Electrophysiological Study in Dogs with Acute Spinal Cord Injury. Korean J. Neurotrauma.

[2] Martin J.H., Quartarone A., Ghilardi M., Boller F. (2022). Neuroplasticity of spinal cord injury and repair. Handbook of Clinical Neurology.

[3] Park S.S., Lee Y.J., Lee S.H., Lee D., Choi K., Kim W.H., Kweon O.K., Han H.J. (2012). Functional recovery after spinal cord injury in dogs treated with a combination of Matrigel and neural-induced adipose-derived mesenchymal Stem cells. Cytotherapy.

[4] Cizkova D., Murgoci A.N., Cubinkova V., Humenik F., Mojzisova Z., Maloveska M., Cizek M., Fournier I., Salzet M. (2020). Spinal Cord Injury: Animal Models, Imaging Tools and the Treatment Strategies. Neurochem. Res..

[5] Nakae A., Nakai K., Yano K., Hosokawa K., Shibata M., Mashimo T. (2011). The animal model of spinal cord injury as an experimental pain model. J. Biomed. Biotechnol..

[6] Ahuja C.S., Nori S., Tetreault L., Wilson J., Kwon B., Harrop J., Choi D., Fehlings M.G. (2017). Traumatic Spinal Cord Injury-Repair and Regeneration. Neurosurgery.

[7] Van Sandt R.L., Welsh C.J., Jeffery N.D., Young C.R., McCreedy D.A., Wright G.A., Boudreau C.E., Levine G.J., Levine J.M. (2022). Circulating neutrophil activation in dogs with naturally occurring spinal cord injury secondary to intervertebral disk herniation. Am. J. Vet. Res..

[8] Nakamoto Y., Tsujimoto G., Ikemoto A., Omori K., Nakamura T. (2021). Pathological changes within two weeks following spinal cord injury in a canine model. Eur. Spine J..

[9] Khan I.U., Yoon Y., Kim A., Jo K.R., Choi K.U., Jung T., Kim N., Son Y., Kim W.H., Kweon O.K. (2018). Improved Healing after the Co-Transplantation of HO-1 and BDNF Overexpressed Mesenchymal Stem Cells in the Subacute Spinal Cord Injury of Dogs. Cell Transplant.

[10] Shi D., He T., Tang W., Li H., Wang C., Zheng M., Hu J., Song X., Ding Y., Chen Y.Y. (2019). Local application of MDL28170-loaded PCL film improves functional recovery by preserving survival of motor neurons after traumatic spinal cord injury. Neurosci. Lett..

[11] Kim W.K., Kim W.H., Kweon O.K., Kang B.J. (2022). Heat-Shock Proteins Can Potentiate the Therapeutic Ability of Cryopreserved Mesenchymal Stem Cells for the Treatment of Acute Spinal Cord Injury in Dogs. Stem Cell Rev..

[12] Song R.B., Basso D.M., da Costa R.C., Fisher L.C., Mo X., Moore S.A. (2016). Adaptation of the Basso-Beattie-Bresnahan locomotor rating scale for use in a clinical model of spinal cord injury in dogs. J. Neurosci. Methods.

[13] Zhang L., Zhuang X., Chen Y., Niu Z., Xia H. (2020). Plasma Erythropoietin, IL-17A, and IFNγ as Potential Biomarkers of Motor Function Recovery in a Canine Model of Spinal Cord Injury. J. Mol. Neurosci..

[14] Laverty P.H., Leskovar A., Breur G.J., Coates J.R., Bergman R.L., Widmer W.R., Toombs J.P., Shapiro S., Borgens R.B. (2004). A preliminary study of intravenous surfactants in paraplegic dogs: Polymer therapy in canine clinical SCI. J. Neurotrauma.

[15] Dalgaard L. (2015). Comparison of minipig, dog, monkey and human drug metabolism and disposition. J. Pharmacol. Toxicol. Methods.

[16] Kim Y., Jo S.H., Kim W.H., Kweon O.K. (2015). Antioxidant and anti-inflammatory effects of intravenously injected adipose derived mesenchymal stem cells in dogs with acute spinal cord injury. Stem Cell Res. Ther..

[17] Metwally E., Zhao G., Zhang Y.Q. (2021). The calcium-dependent protease calpain in neuronal remodeling and neurodegeneration. Trends Neurosci..

[18] Ono Y., Saido T.C., Sorimachi H. (2016). Calpain research for drug discovery: Challenges and potential. Nat. Rev. Drug Discov..

[19] Yu C.G., Bondada V., Joshi A., Reneer D.V., Telling G.C., Saatman K.E., Geddes J.W. (2020). Calpastatin Overexpression Protects against Excitotoxic Hippocampal Injury and Traumatic Spinal Cord Injury. J. Neurotrauma.

[20] Li Y., Gong Z.H., Sheng L., Gong Y.T., Tan X.Y., Li W.M., Dong D.L., Yang B.F., Fu S.B., Xue H.J. (2009). Anti-apoptotic effects of a calpain inhibitor on cardiomyocytes in a canine rapid atrial fibrillation model. Cardiovasc. Drugs Ther..

[21] Kimura T., Ishiguro K., Hisanaga S. (2014). Physiological and pathological phosphorylation of tau by Cdk5. Front. Mol. Neurosci..

[22] Hung K.S., Hwang S.L., Liang C.L., Chen Y.J., Lee T.H., Liu J.K., Howng S.L., Wang C.H. (2005). Calpain inhibitor inhibits p35-p25-Cdk5 activation, decreases tau hyperphosphorylation, and improves neurological function after spinal cord hemisection in rats. J. Neuropathol. Exp. Neurol..

[23] Baltin M.E., Sabirova D.E., Kiseleva E.I., Kamalov M.I., Abdullin T.I., Petrova N.V., Ahmetov N.F., Sachenkov O.A., Baltina T.V., Lavrov I.A. (2021). Comparison of systemic and localized carrier-mediated delivery of methylprednisolone succinate for treatment of acute spinal cord injury. Exp. Brain Res..

[24] Khan M.F., Burks S.S., Al-Khayat H., Levi A.D. (2014). The effect of steroids on the incidence of gastrointestinal hemorrhage after spinal cord injury: A case-controlled study. Spinal Cord..

[25] Hurlbert R.J. (2014). Methylprednisolone for the treatment of acute spinal cord injury: Point. Neurosurgery.

[26] Duan K., Liu S., Yi Z., Liu H., Li J., Shi J., Ji L., Xu B., Zhang X., Zhang W. (2021). S100-β aggravates spinal cord injury via activation of M1 macrophage phenotype. J. Musculoskelet. Neuronal Interact..

[27] Mandwie M., Piper J.A., Gorrie C.A., Keay K.A., Musumeci G., Al-Badri G., Castorina A. (2022). Rapid GFAP and Iba1 expression changes in the female rat brain following spinal cord injury. Neural Regen. Res..

[28] Moon S.M., Kim W., Chung J.Y., Im W., Yoo D.Y., Jung H.Y., Won M.H., Choi J.H., Hwang I.K. (2014). Neuroprotective Effects of Adipose-Derived Stem Cells Are Maintained for 3 Weeks against Ischemic Damage in the Rabbit Spinal Cord. BioMed Res. Int..

[29] Sribnick E.A., Matzelle D.D., Banik N.L., Ray S.K. (2007). Direct evidence for calpain involvement in apoptotic death of neurons in spinal cord injury in rats and neuroprotection with calpain inhibitor. Neurochem. Res..

[30] Lee M.S., Kwon Y.T., Li M., Peng J., Friedlander R.M., Tsai L.H. (2000). Neurotoxicity induces cleavage of p35 to p25 by calpain. Nature.

[31] Kurbatskaya K., Phillips E.C., Croft C.L., Dentoni G., Hughes M.M., Wade M.A., Al-Sarraj S., Troakes C., O’Neill M.J., Perez-Nievas B.G. (2016). Upregulation of calpain activity precedes tau phosphorylation and loss of synaptic proteins in Alzheimer’s disease brain. Acta Neuropathol. Commun..

[32] Caprelli M.T., Mothe A.J., Tator C.H. (2018). Hyperphosphorylated Tau as a Novel Biomarker for Traumatic Axonal Injury in the Spinal Cord. J. Neurotrauma..

[33] Nishida H., Tanaka H., Kitamura M., Inaba T., Nakayama M. (2016). Methylprednisolone sodium succinate reduces spinal cord swelling but does not affect recovery of dogs with surgically treated thoracolumbar intervertebral disk herniation. Jpn. J. Vet. Res..

[34] Yang J.W., Jeong S.M., Seo K.M., Nam T.C. (2003). Effects of corticosteroid and electroacupuncture on experimental spinal cord injury in dogs. J. Vet. Sci..

[35] Cheriyan T., Ryan D.J., Weinreb J.H., Cheriyan J., Paul J.C., Lafage V., Kirsch T., Errico T.J. (2014). Spinal cord injury models: A review. Spinal Cord..

[36] Lee J.H., Choi C.B., Chung D.J., Kang E.H., Chang H.S., Hwang S.H., Han H., Choe B.Y., Sur J.H., Lee S.Y. (2008). Development of an improved canine model of percutaneous spinal cord compression injury by balloon catheter. J. Neurosci. Methods.

[37] Fukuda S., Nakamura T., Kishigami Y., Endo K., Azuma T., Fujikawa T., Tsutsumi S., Shimizu Y. (2005). New canine spinal cord injury model free from laminectomy. Brain Res. Brain Res. Protoc..

[38] Ryu H.H., Lim J.H., Byeon Y.E., Park J.R., Seo M.S., Lee Y.W., Kim W.H., Kang K.S., Kweon O.K. (2009). Functional recovery and neural differentiation after transplantation of allogenic adipose-derived stem cells in a canine model of acute spinal cord injury. J. Vet. Sci..

[39] Purdy P.D., Duong R.T., White C.L., Baer D.L., Reichard R.R., Pride G.L., Adams C., Miller S., Hladik C.L., Yetkin Z. (2003). Percutaneous translumbar spinal cord compression injury in a dog model that uses angioplasty balloons: MR imaging and histopathologic findings. Am. J. Neuroradiol..

[40] Purdy P.D., White C.L., Baer D.L., Frawley W.H., Reichard R.R., Pride G.L., Adams C., Miller S., Hladik C.L., Yetkin Z. (2004). Percutaneous translumbar spinal cord compression injury in dogs from an angioplasty balloon: MR and histopathologic changes with balloon sizes and compression times. Am. J. Neuroradiol..

[41] Haldipur N., Tan P., Katory M., Singh S. (2002). A safe method of retrograde passage of fogarty embolectomy catheter through difficult iliac arteries. Eur. J. Vasc. Endovasc. Surg..

[42] Molano Mdel R., Broton J.G., Bean J.A., Calancie B. (2002). Complications associated with the prophylactic use of methylprednisolone during surgical stabilization after spinal cord injury. J. Neurosurg..

[43] Plantier V., Sanchez-Brualla I., Dingu N., Brocard C., Liabeuf S., Gackière F., Brocard F. (2019). Calpain fosters the hyperexcitability of motoneurons after spinal cord injury and leads to spasticity. eLife.

[44] Ray S.K., Hogan E.L., Banik N.L. (2003). Calpain in the pathophysiology of spinal cord injury: Neuroprotection with calpain inhibitors. Brain Res. Brain Res. Rev..

[45] Jia Z., Zhu H., Li J., Wang X., Misra H., Li Y. (2012). Oxidative stress in spinal cord injury and antioxidant-based intervention. Spinal Cord..

[46] Hall E.D., Wang J.A., Bosken J.M., Singh I.N. (2016). Lipid peroxidation in brain or spinal cord mitochondria after injury. J. Bioenerg. Biomembr..

[47] Dimitrijevic M.R., Danner S.M., Mayr W. (2015). Neurocontrol of Movement in Humans With Spinal Cord Injury. Artif. Organs..

[48] Park E.H., White G.A., Tieber L.M. (2012). Mechanisms of injury and emergency care of acute spinal cord injury in dogs and cats. J. Vet. Emerg. Crit. Care.

[49] Banik N.L., Matzelle D., Terry E., Hogan E.L. (1997). A new mechanism of methylprednisolone and other corticosteroids action demonstrated in vitro: Inhibition of a proteinase (calpain) prevents myelin and cytoskeletal protein degradation. Brain Res..

[50] Ray S.K., Wilford G.G., Matzelle D.C., Hogan E.L., Banik N.L. (1999). Calpeptin and methylprednisolone inhibit apoptosis in rat spinal cord injury. Ann. N. Y. Acad. Sci..

[51] Mills C.D., Xu G.Y., McAdoo D.J., Hulsebosch C.E. (2001). Involvement of metabotropic glutamate receptors in excitatory amino acid and GABA release following spinal cord injury in rat. J Neurochem..

[52] Wang C., Shi D., Song X., Chen Y., Wang L., Zhang X. (2016). Calpain inhibitor attenuates ER stress-induced apoptosis in injured spinal cord after bone mesenchymal stem cells transplantation. Neurochem. Int..

[53] Zhang Z., Huang Z., Dai H., Wei L., Sun S., Gao F. (2015). Therapeutic Efficacy of E-64-d, a Selective Calpain Inhibitor, in Experimental Acute Spinal Cord Injury. Biomed. Res. Int..

[54] Wang K.K., Nath R., Posner A., Raser K.J., Buroker-Kilgore M., Hajimohammadreza I., Probert A.W., Marcoux F.W., Ye Q., Takano E. (1996). An alpha-mercaptoacrylic acid derivative is a selective nonpeptide cell-permeable calpain inhibitor and is neuroprotective. Proc. Natl. Acad. Sci. USA.

[55] Nathan C., Ding A. (2010). Nonresolving inflammation. Cell..

[56] Vidal P.M., Lemmens E., Geboes L., Vangansewinkel T., Nelissen S., Hendrix S. (2013). Late blocking of peripheral TNF-α is ineffective after spinal cord injury in mice. Immunobiology.

[57] Milatovic D., Zaja-Milatovic S., Brockett M.M., Breyer R.M., Aschner M., Montine T.J., Gupta R. (2022). Neuroinflammation and oxidative injury in developmental neurotoxicity. Reproductive and Developmental Toxicology.

[58] Yu L., Qian J. (2020). Dihydrotanshinone I Alleviates Spinal Cord Injury via Suppressing Inflammatory Response, Oxidative Stress and Apoptosis in Rats. Med. Sci. Monit..

[59] Spitzbarth I., Bock P., Haist V., Stein V.M., Tipold A., Wewetzer K., Baumgärtner W., Beineke A. (2011). Prominent microglial activation in the early proinflammatory immune response in naturally occurring canine spinal cord injury. J. Neuropathol. Exp. Neurol..

[60] Pusterla N., Wilson W.D., Conrad P.A., Mapes S., Leutenegger C.M. (2006). Comparative analysis of cytokine gene expression in cerebrospinal fluid of horses without neurologic signs or with selected neurologic disorders. Am. J. Vet. Res..

[61] Bazinet R.P., Layé S. (2014). Polyunsaturated fatty acids and their metabolites in brain function and disease. Nat. Rev. Neurosci..

[62] Shohami E., Beit-Yannai E., Horowitz M., Kohen R. (1997). Oxidative stress in closed-head injury: Brain antioxidant capacity as an indicator of functional outcome. J. Cereb. Blood Flow Metab..

[63] Naik A.K., Tandan S.K., Dudhgaonkar S.P., Jadhav S.H., Kataria M., Prakash V.R., Kumar D. (2006). Role of oxidative stress in pathophysiology of peripheral neuropathy and modulation by N-acetyl-L-cysteine in rats. Eur. J. Pain..

[64] Morsy M.D., Mostafa O.A., Hassan W.N. (2010). A potential protective effect of alpha-tocopherol on vascular complication in spinal cord reperfusion injury in rats. J. Biomed. Sci..

[65] Marquis A., Packer R.A., Borgens R.B., Duerstock B.S. (2015). Increase in oxidative stress biomarkers in dogs with ascending-descending myelomalacia following spinal cord injury. J. Neurol. Sci..

[66] Fu D., Liu H., Li S., Chen L., Yao J. (2017). Antioxidative and Antiapoptotic Effects of Delta-Opioid Peptide [D-Ala(2), D-Leu(5)] Enkephalin on Spinal Cord Ischemia-Reperfusion Injury in Rabbits. Front. Neurosci..

[67] Zirak A., Soleimani M., Jameie S.B., Abdollahifar M.A., Fadaei Fathabadi F., Hassanzadeh S., Esmaeilzadeh E., Farjoo M.H., Norouzian M. (2021). Related Fluoxetine and Methylprednisolone Changes of TNF-α and IL-6 Expression in The Hypothyroidism Rat Model of Spinal Cord Injury. Cell J..

[68] Can M., Gul S., Bektas S., Hanci V., Acikgoz S. (2009). Effects of dexmedetomidine or methylprednisolone on inflammatory responses in spinal cord injury. Acta Anaesthesiol. Scand..

[69] Chen Y.S., Tseng F.Y., Tan C.T., Lin-Shiau S.Y., Hsu C.J. (2008). Effects of methylprednisolone on nitric oxide formation and survival of facial motor neurons after axotomy. Brain Res..

[70] Cavus G., Altas M., Aras M., Ozgür T., Serarslan Y., Yilmaz N., Sefil F., Ulutas K.T. (2014). Effects of montelukast and methylprednisolone on experimental spinal cord injury in rats. Eur. Rev. Med. Pharmacol. Sci..

[71] Keles I., Bozkurt M.F., Aglamis E., Fidan A.F., Ceylan C., Karalar M., Coban S., Denk B., Buyukokuroglu M.E. (2019). Protective effects of dantrolene and methylprednisolone against spinal cord injury-induced early oxidative damage in rabbit bladder: A comparative experimental study. Adv. Clin. Exp. Med..

[72] Liu D., Yan Z., Minshall R.D., Schwartz D.E., Chen Y., Hu G. (2012). Activation of calpains mediates early lung neutrophilic inflammation in ventilator-induced lung injury. Am. J. Physiol. Lung Cell Mol. Physiol..

[73] Ni R., Zheng D., Xiong S., Hill D.J., Sun T., Gardiner R.B., Fan G.C., Lu Y., Abel E.D., Greer P.A. (2016). Mitochondrial Calpain-1 Disrupts ATP Synthase and Induces Superoxide Generation in Type 1 Diabetic Hearts: A Novel Mechanism Contributing to Diabetic Cardiomyopathy. Diabetes.

[74] Chen S.X., Wang S.K., Yao P.W., Liao G.J., Na X.D., Li Y.Y., Zeng W.A., Liu X.G., Zang Y. (2018). Early CALP2 expression and microglial activation are potential inducers of spinal IL-6 up-regulation and bilateral pain following motor nerve injury. J. Neurochem..

[75] Faul F., Erdfelder E., Lang A.G., Buchner A. (2007). G*Power 3: A flexible statistical power analysis program for the social, behavioral, and biomedical sciences. Behav. Res. Methods.

[76] Council N.R. (2006). Nutrient Requirements of Dogs and Cats.

[77] Clarke K.W., Trim C.M., Hall L.W., Clarke K.W., Trim C.M., Hall L.W. (2014). Anaesthesia of the dog. Veterinary Anaesthesia.

[78] Lee S.H., Kim Y., Rhew D., Kim A., Jo K.R., Yoon Y., Choi K.U., Jung T., Kim W.H., Kweon O.K. (2017). Effect of canine mesenchymal stromal cells overexpressing heme oxygenase-1 in spinal cord injury. J. Vet. Sci..

[79] Kerwin S.C., Levine J.M., Mankin J.M., Johnston S.A., Tobias K.M. (2018). Thoracolumbar Vertebral Column. Veterinary Surgery: Small Animal.

[80] David S., López-Vales R., Wee Yong V., Verhaagen J., McDonald J.W. (2012). Harmful and beneficial effects of inflammation after spinal cord injury: Potential therapeutic implications. Handbook of Clinical Neurology.

[81] Freeman L.M., Rush J.E., Milbury P.E., Blumberg J.B. (2005). Antioxidant status and biomarkers of oxidative stress in dogs with congestive heart failure. J. Vet. Intern. Med..

[82] Miranda K.M., Espey M.G., Wink D.A. (2001). A rapid, simple spectrophotometric method for simultaneous detection of nitrate and nitrite. Nitric Oxide.

[83] Zhang X.H., Pei G.X., Wei K.H., Zhou X.J. (2002). Dynamic changes of SOD and MDA in canine limbs with gunshot wound in hot and humid environment: An experimental study. Di Yi Jun Yi Da Xue Xue Bao.

[84] Merbl Y., Sommer A., Chai O., Aroch I., Zimmerman G., Friedman A., Soreq H., Shamir M.H. (2014). Tumor necrosis factor-α and interleukin-6 concentrations in cerebrospinal fluid of dogs after seizures. J. Vet. Intern. Med..

[85] Poncelet L., Michaux C., Balligand M. (1993). Somatosensory potentials in dogs with naturally acquired thoracolumbar spinal cord disease. Am. J. Vet. Res..

[86] Close B., Banister K., Baumans V., Bernoth E.M., Bromage N., Bunyan J., Erhardt W., Flecknell P., Gregory N., Hackbarth H. (1997). Recommendations for euthanasia of experimental animals: Part 2. Lab. Anim..

[87] Layton C., Bancroft J.D., Suvarna S.K., Suvarna S.K., Layton C., Bancroft J.D. (2019). Fixation of tissues. Bancroft’s Theory and Practice of Histological Techniques.

[88] Deng W.S., Liu X.Y., Ma K., Liang B., Liu Y.F., Wang R.J., Chen X.Y., Zhang S. (2021). Recovery of motor function in rats with complete spinal cord injury following implantation of collagen/silk fibroin scaffold combined with human umbilical cord-mesenchymal stem cells. Rev. Da Assoc. Médica Bras..

[89] Carriel V., Campos A., Alaminos M., Raimondo S., Geuna S. (2017). Staining Methods for Normal and Regenerative Myelin in the Nervous System. Methods Mol. Biol..

[90] Buchwalow I.B., Böcker W. (2010). Background Staining, Autofluorescence and Blocking Steps. Immunohistochemistry: Basics and Methods.

[91] Schindelin J., Arganda-Carreras I., Frise E., Kaynig V., Longair M., Pietzsch T., Preibisch S., Rueden C., Saalfeld S., Schmid B. (2012). Fiji: An open-source platform for biological-image analysis. Nat. Methods.

